# Supplementary material for: Nonlinear Chiral-like State Transfer realized with a minimal set of parameters
Source: Nat Commun. 2025 Jul 1;16:5844. doi: 10.1038/s41467-025-61372-2 (PMC12216736; doi:10.1038/s41467-025-61372-2)
Supplement: Supplementary file 1 — Supplementary Information [file 41467_2025_61372_MOESM1_ESM.pdf]

Supplementary Information of

"Nonlinear Chiral-like State Transfer realized with a minimal set of parameters"

Kai Bai,[1] Chen Lin,[1] Tao Liu,[1] Jia-Zheng Li,[1] Xin Lyu,[1] and Meng Xiao,[1,2]

[1]Key Laboratory of Artificial Micro- and Nano-structures of Ministry of Education and School of Physics and Technology, Wuhan University, Wuhan 430072, China

[2]Wuhan Institute of Quantum Technology, Wuhan 430206, China

Corresponding Email [*]: phmxiao@whu.edu.cn

Supplementary Note 1: Conceptual evolution of CST.

In this section, we outline the conceptual evolution of CST. The quantum adiabatic theorem, a seminal result in quantum mechanics, states that for an infinitely slow parametric perturbation there is no possibility of a quantum jump. With the rapidly growing interest in non-Hermitian systems, Ref. [1] first proposed that when system parameters are adiabatically varied along a loop enclosing an exceptional point (EP), nonadiabatic transitions (NATs) inevitably occur. As a result, for any initial state that is a linear combination of the two eigenstates, the final state after one encircling cycle depends solely on the direction of encirclement—clockwise or counterclockwise. This dependence of the final state on the encircling direction is referred to as CST. Since then, this phenomenon has attracted considerable attention from both theoretical and experimental communities—not only due to its physical elegance and counterintuitive nature, but also because of its great potential in quantum information processing, quantum communication, and on-chip photonic devices.

In recent years, with the introduction of new concepts, the definition of CST in non-Hermitian systems has continued to evolve. For instance, the parameter trajectories that give rise to this peculiar dependence of the final state have been extended to include those that do not encircle an EP[2,3], as well as open paths that do not form a closed loop[4–6]. In particular, the path proposed in Ref. [6] is reduced to a straight line with an additional single point in the parameter space. It is worth noting that, for an open path, the notion of clockwise or counterclockwise evolution is no longer well-defined in the conventional sense. Nevertheless, this phenomenon continues to be referred to as CST in the literature. In essence, the core mechanism of CST is the controllable NATs during the parameter sweep.

To more clearly highlight the similarities and differences between EP-based CST and NEP- based NCST, we have included Table S1 for comparison.

Parameter steering property	EP-related CST	NEP-related NCST

Adiabatic; Closed loop including EP/NEP.	Final state is determined solely by the direction[1].	Final state is determined solely by the direction[7].
Adiabatic; Closed loop excluding EP/NEP.	Final state is determined by the direction and trajectory[2,3].	Final state is determined by the direction and trajectory.
Open loop.	Final state is trajectory dependent [4–6].	Final state is determined by trajectory and evolution time.
Physical mechanism.	Landscape of eigen-spectrum and non-adiabatic jump.	Landscape of eigen-spectrum and attractor property.

TABLE S1. Similarities and differences between CST and NCST.

Supplementary Note 2: CSTs realized through adiabatically encirclement of a NEP.

In this section, we detail the approach to achieving chiral state transfers (CSTs) by adiabatically encircling a nonlinear exceptional point (NEP) in the parameter space. The model is replotted in Fig. S1(a), which consists of two resonant modes with resonance frequencies ω_A and ω_B, coupling rate κ, linear loss l_B, and nonlinear gain $g_A(|\psi_A|)$. The corresponding nonlinear Schrödinger equation is

$$\begin{pmatrix} \omega_A + ig_A(|\psi_A|) & \kappa \\ \kappa & \omega_B - il_B \end{pmatrix} \begin{pmatrix} \psi_A(t) \\ \psi_B(t) \end{pmatrix} = \omega \begin{pmatrix} \psi_A \\ \psi_B \end{pmatrix}, \tag{S1}$$

where ω is the eigenfrequency, $\psi_A \equiv |\psi_A| e^{i\theta_A}$ and $\psi_B \equiv |\psi_B| e^{i\theta_B}$ representing the field of the red (left) and blue (right) resonators A and B, respectively. As had been derived in Refs.[8–11], we find the steady states of the nonlinear Hamiltonian in Eq. (S1) by letting $g_A(|\psi_A|)$ be a free parameter to tune. In optics, a commonly used gain saturation model is $g_A(|\psi_A|) = \Gamma/(1 + |\psi_A|^2) - \gamma_0$ with Γ representing the pump strength and γ_0 representing the intrinsic loss [12–14]. The gain model in the circuit of our experiment is discussed in the following sections. The strength of $g(|\psi_A|)$

corresponding to a steady state solution defines the saturated gain level g_s that satisfies

$$-2\kappa - l_B(\omega_A - \omega) + g_s(\omega_B - \omega) = 0. \qquad \text{(S2)}$$

And the steady state frequency ω is determined by the following real coefficient polynomial:

$$p(\omega) = (\omega_A - \omega)(\omega_B - \omega)^2 + l_B^2(\omega_A - \omega) - \kappa^2(\omega_B - \omega) = 0. \qquad \text{(S3)}$$

Here, the gain saturation form $g(|\psi_A|)$ is irrelevant for $p(\omega)$ as long as Eq. (S2) can be satisfied [11]. With the self-consistent eigenfrequencies ω and saturated gains g_s at hand, we can calculate the self-consistent eigenmodes $(\psi_A, \psi_B)^T$ from Eq. (S1). For simplicity, all the parameters are normalized by κ, and we set ω_A equal to 0 since any global frequency shift is irrelevant. At ω_B=0 and $l_B = 1$, a NEP_3 is achieved. To clearly illustrate the properties in the vicinity of this NEP_3, δx represents the perturbation along the l_B direction (i.e., $1 + \delta x$), and δy represents the perturbation along the ω_A direction (i.e., $0 + \delta y$). Figure S1(b-d) shows the corresponding phase differences $\theta_{B-A} \equiv (\theta_B - \theta_A)$, frequencies ω, and amplitude ratios $r \equiv |\psi_A/\psi_B|$ for the self-consistent eigenmodes, respectively.

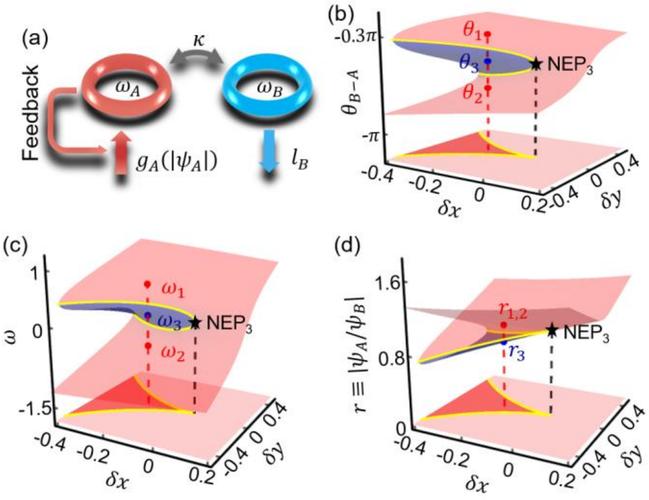

FIG. S1. (a) Schematic of our nonlinear model. Phase differences θ_{B-A} (b), frequencies ω (c), and amplitude ratios $r \equiv |\psi_A/\psi_B|$ (d) of the steady modes versus δx and δy in the parameter space. The light red and light blue regions represent stable and unstable modes, respectively. On the $\delta x - \delta y$ plane, the light red and red color represent the parameter regions with monostable and bistable modes, respectively. The

yellow lines represent nonlinear exceptional arcs (NEAs) consisting entirely of NEP_2s, which also serve as boundaries between the monostable and bistable region. At the intersection of these two NEAs, the black star represents the NEP_3 ($\delta x = \delta y = 0$), where two stable modes and one unstable mode coalesce.

Now, we numerically analyze the CST when slowly encircling this NEP_3 in the $\delta x - \delta y$ parameter space. The system's dynamics is governed by the time-dependent nonlinear equation:

$$i\frac{d}{dt}\begin{pmatrix}\psi_A\\ \psi_B\end{pmatrix} = \begin{pmatrix}\omega_A + ig_A(|\psi_A|) & \kappa\\ \kappa & \omega_B - il_B\end{pmatrix}\begin{pmatrix}\psi_A(t)\\ \psi_B(t)\end{pmatrix}. \quad \text{(S4)}$$

For demonstration purposes, the trajectory of the encirclement (loop) is defined as $\omega_A \equiv 0 + \delta y(t)$ and $l_B \equiv 1 + \delta x(t)$. We set $\delta x(t) = -\beta\cos(\tau\, 2\,\pi\, t/T)$ and $\delta y(t) = -\beta\sin(\tau\, 2\,\pi\, t/T)$ with β representing the radius of the loop, T denoting the cycle period and $\tau = \pm 1$ for the winding direction ($\tau = +1$ for anticlockwise and $\tau = -1$ for clockwise). We assume the system starts at a higher phase difference state, as marked by the bold green arrows [see Fig. S2(a, c)]. Along the anticlockwise direction [Fig. S2(a)], the state experiences a nonadiabatic transition at p_1 and evolves to the same state from where it starts (the open black square), i.e., the higher phase difference state. In contrast, along the clockwise direction [Fig. S2(c)], the state stays on the stable steady-state surface at all times, evolving adiabatically to a lower phase differences state (the open black square). Figures S2(b) and S2(d) show the corresponding evolution of $\delta x(t)$, $\delta y(t)$, $|\psi_{A,B}|$, and θ_{B-A} for the clockwise and anticlockwise encircling directions, respectively. Here the x axis covers one period with $T = 3000$, and the encirclement can be regarded as adiabatic. These evolutions elucidate the fact that, provided the circling process is slow enough, dynamical encirclement of a NEP_3 in the parameter space can lead to CSTs.

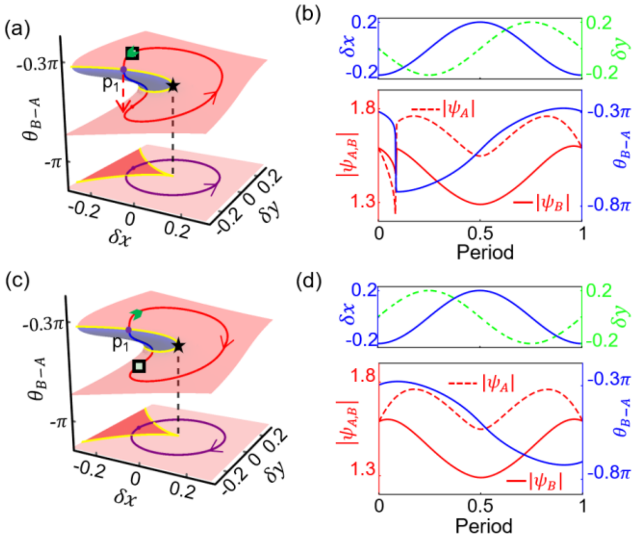

FIG. S2. Given a higher phase difference initial state [marked by the bold green arrow in (a, c)], the state experiences a nonadiabatic transition at p_1, and ends with the same initial state (marked by the open black square) in the counterclockwise direction (a, b); In contrast, along the clockwise direction (c, d), the state evolves adiabatically and ends at a lower phase difference final state (the open black square).

Supplementary Note 3:NCSTs at different parameter steering speeds.

In this section, we detail the influence of steering speed on NCST. The NCST between bistable modes arises from the contribution of the attractor. When the system evolves in different directions along a given trajectory, the instantaneous states fall into distinct basins of attraction, thereby enabling the realization of NCST. Therefore, NCST can be achieved without requiring adiabatic parameter steering. In other words, the steering speed can be substantially increased, such that the system—except at S_i—no longer has sufficient time to fully evolve toward its corresponding stable states.

To unveil this fact more explicitly, Fig. S3 shows a single-cycle parametric loop centered at the NEP_3. During an adiabatically dynamical encirclement of the NEP_3, the evolved state is always restricted to the stable state [see Fig. S3(b, e)]. Along the clockwise direction, the state experiences a nonadiabatic transition at t_1 and evolves to the same lower-phase-difference state from where it starts [see Fig. S3(b)]. Along the anticlockwise direction, the state stays on the steady stable state surface at all times, evolving adiabatically from a lower-phase-difference state to a higher-phase-difference

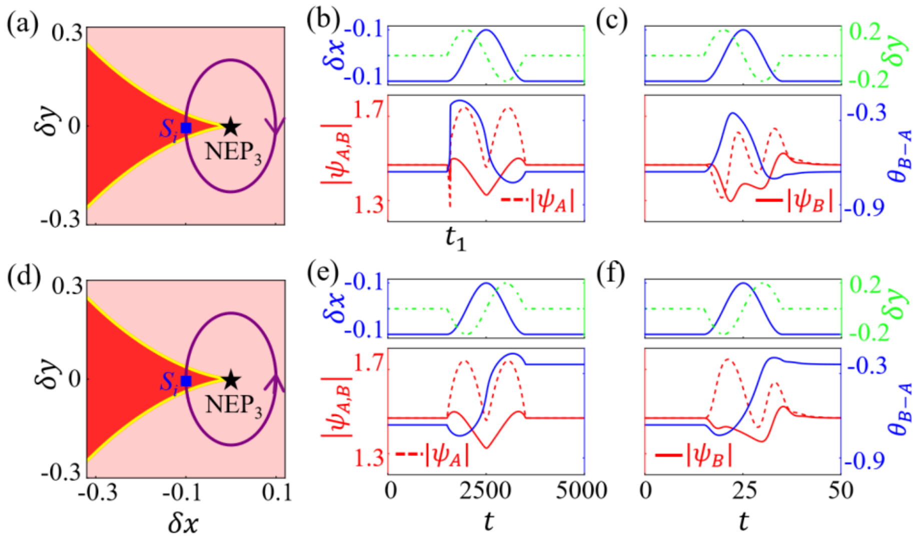

FIG. S3. (a-c), Along the clockwise direction, the evolution trajectory of steering parameters in the parameter space (a) and of the states in the phase space for different steering speeds (b, c). (d-f), The same as (a-c), but for anticlockwise encirclement. Here, the initial state is selected as the lower-phase-difference stable state. In (b, e), the instantaneous state is restricted to the stable state except at the point (t_1 in b) where nonadiabatic transitions occur. In contrast, in (c, f), the instantaneous state deviates from the stable state for most of the evolution. The other unspecified parameters used are the same as those in Fig. 2.

state [see Fig. S3(e)]. Here the encircling period $T = 5000$, and the encirclement can be regarded as adiabatic. These evolutions elucidate the fact that, provided the circling process is slow enough, dynamical encirclement of a NEP$_3$ in the parameter space can lead to CSTs and that the evolved state is restricted to the stable state except at the points where nonadiabatic transitions occur. However, the NCST discussed here arises from the contribution of the attractor, the steering speed can be substantially increased. For instance, we can reduce the steering period from T=5000 to T=50. Figure S3 (c, f) shows the corresponding evolution trajectories of the states in phase space (see the lower panels), which differ significantly from those in Fig. S3(b, e). Thus, the instantaneous state deviates from the stable state for most of the evolution. In other words, the system—except at S_i—no longer has sufficient time to fully evolve toward its corresponding stable state.

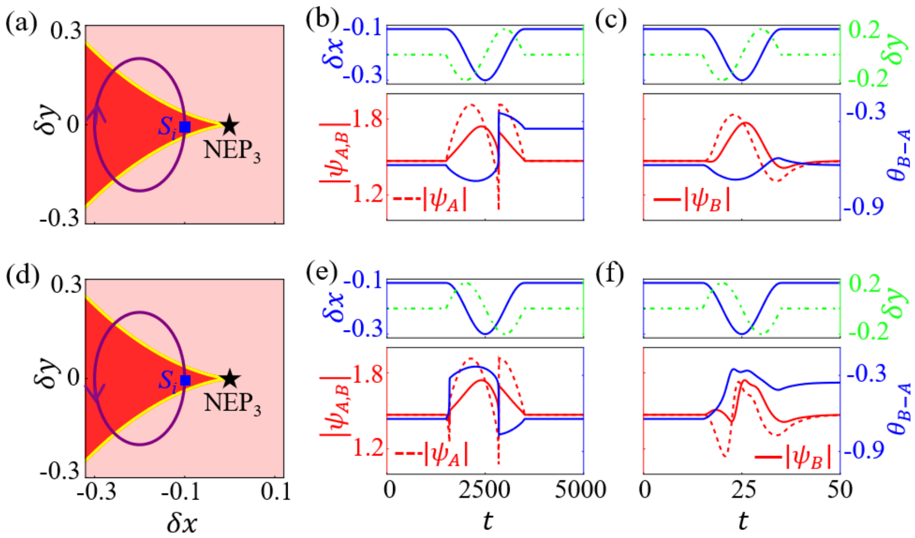

FIG. S4. “Reversal” of chirality at different steering speeds. The same as Fig. R2, but now the NEP_3 is not enclosed by the parametric loop.

For some configurations of the encirclement loops, the speed of the parameter steering process greatly influences the outcome, which may result in a “reversal” of chirality (see Fig. S4) or disappearance of the chiral behavior (see Fig. S5). Figure S4 shows a single-cycle parametric loop that does not enclose the NEP_3. Along this path, both adiabatic driving [see Fig. S4(b, e) with T=5000] and fast steering [see Fig. S4(c, f) with T=50] give rise to NCSTs. However, a closer comparison between Figs. S4(b) and S4(c), as well as S4(e) and S4(f), reveals that the final evolved states differ, despite the same parametric path being followed. In Fig. S5, the trajectory of the steering process is simplified to three distinct parameter points $\{S_i, S_1, S_2\}$. When the steering period $T = 50$, NCST is still observed. However, as the steering period is further reduced to $T = 5$, the chiral behavior is destroyed, despite the same parametric path being followed.

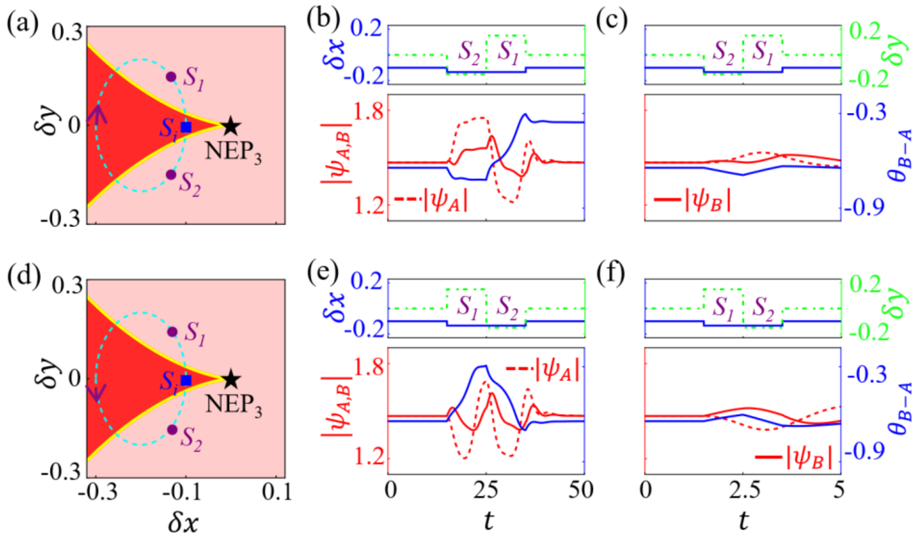

FIG. S5. Disappearance of the chiral behavior. The same as Fig. S4, but the trajectory of the parameter steering process is simplified to three distinct points $\{\mathrm{S}_i, \mathrm{S}_1, \mathrm{S}_2\}$.

Supplementary Note 4:NCSTs achieved by varying only a single component.

In this section, we detail that NCSTs can be achieved by varying only a single component, which further simplifies the experimental implementation. In the tight-binding model used in our work, changing a single component corresponds to a straight line in the parameter space. However, in practical experimental settings, each external tuning knob typically affects multiple parameters of the tight-binding model. For instance, in our circuit implementation, adjusting a component such as the capacitance C_0 typically leads to simultaneous changes in both the resonant frequencies of the cavities and the effective loss rates in the equivalent tight-binding model [see Eq. (4) in the main text]. In coupled optical waveguides[15,16] and microcavities[17,18], the tuning of the resonance frequency can also cause a change in the coupling strength. Thanks to the absence of strict constraints on $\{S_1, S_2\}$, NCST can be realized by varying only a single external tuning knob. For demonstration purposes, we systematically vary the positions of $\{S_1, S_2\}$: initially placing them to the right of the NEP3 [i.e., $\delta_x > 0$, see Figs. S6(a)], moving them to the region between S_i and the NEP3 [i.e., $-0.1 < \delta_x < 0$, see Figs.

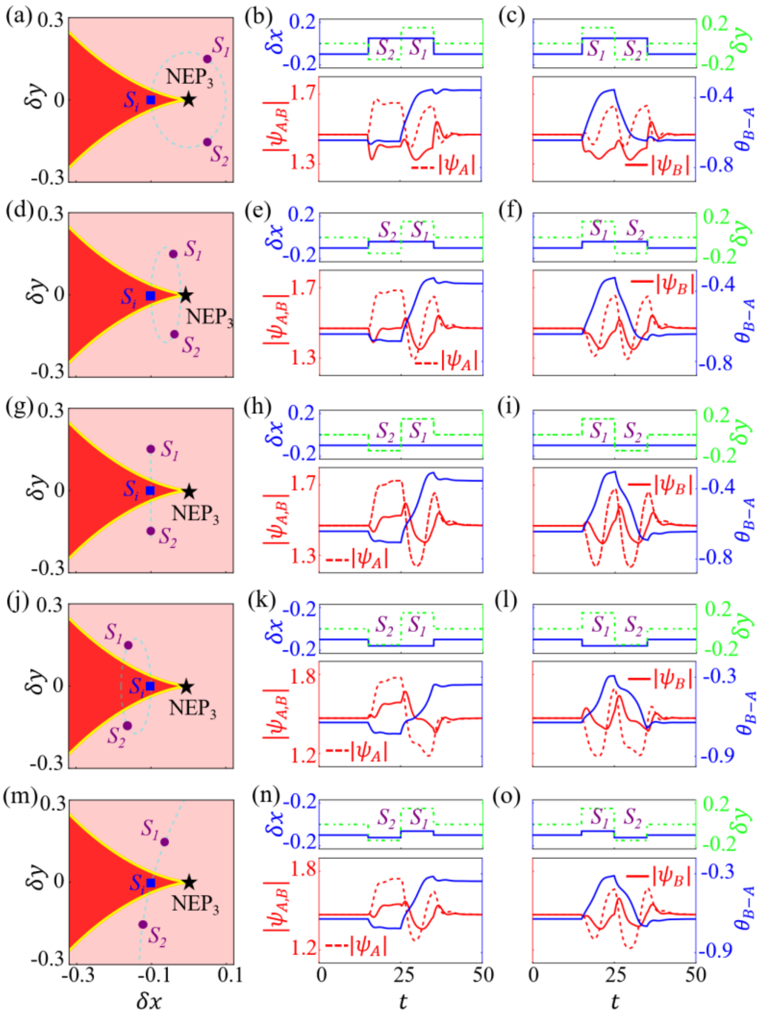

FIG. S6. NCSTs under different configurations. To achieve NCST, the initial point S_i must lie within the bistable region; however, the points $\{S_1, S_2\}$ are not subject to strict constraints. The first column illustrates different configurations of $\{S_1, S_2\}$; while the second and third columns show the corresponding evolutions of the parameters in parameter space (upper panels) and the system states in phase space (lower panels), respectively. In (a, d, g, j), we fix $\delta y = 0.15$ and -0.15 for S_1 and S_2, respectively. The values of δx for $\{S_1, S_2\}$ are set to be 0.05 in (a), -0.04 in (d), -0.1 in (g) and -0.16 in (j). In the tight-binding model used in our work, NCST can be realized by varying only δy. In the experimental implementation, δx and δy are generally not independent. In (m), we assume $\delta x = -0.1 + 0.2\,\eta + 0.3\,\eta^2$ and $\delta y = \eta$, with $\eta = 0.15$ for S_1 and $\eta = -0.15$ for S_2. Other parameters used are the same as those in Fig. 2.

S6(d)], then aligning them with S_i along a vertical line [i.e., $\delta_x = -0.1$, see Figs.S6(g)], and finally placing them to the left of S_i [i.e., $\delta_x < -0.1$, see Figs. S6(j)]. The cyan dashed lines indicate that all three points—$\{S_i, S_1, S_2\}$—can be reached by varying only a single parameter. Figures S6(b, c), S6(e, f), S6(h, i), and S6(k, l) show the corresponding parameter and state evolution for each case. Clearly, this dependence of the final state is faithfully preserved during the parameter variation. Furthermore, we randomly choose $\delta x = -0.1 + 0.2\,\eta + 0.3\,\eta^2$ and $\delta y = \eta$, where η corresponds to the variation of a single physical component. Figure S6(m-o) shows that this dependence of the final state is also faithfully preserved. Note here, the three points $\{S_i, S_1, S_2\}$ are not on the same straight line in the parameter space though we only tune a single knob, i.e., η.

Supplementary Note 5: Reliable predictability of NCSTs in non-adiabatic regimes.

In this section, we detail reliable predictability of NCSTs in non-adiabatic regimes. From a theoretical and conceptual standpoint, adiabatic CST schemes based on EP or NEP encirclement are highly appealing due to their simplicity and full predictability, as the final outcome is determined solely by the encircling direction. However, from an experimental perspective, such adiabatic parametric steering processes typically requires long evolution times — which translate to bulky devices in waveguide systems—and extremely complex setups to suppress noise accumulation. There is a general trade-off—relevant to both CST and NCST—between the theoretical preference for adiabatic evolution with full predictability, and the experimental demand for faster evolution to reduce setup complexity and broaden practical applicability. This trade-off has already been taken into account in the experimental design of EP-based CST demonstration[3,19]. Reducing the evolution time is desirable, provided that CST can still be stably achieved. Although such schemes inevitably reduce the level of predictability to some extent, as long as the final outcome remains robust within the bounds of experimental uncertainty, the process can still be considered effectively predictable and of practical relevance.

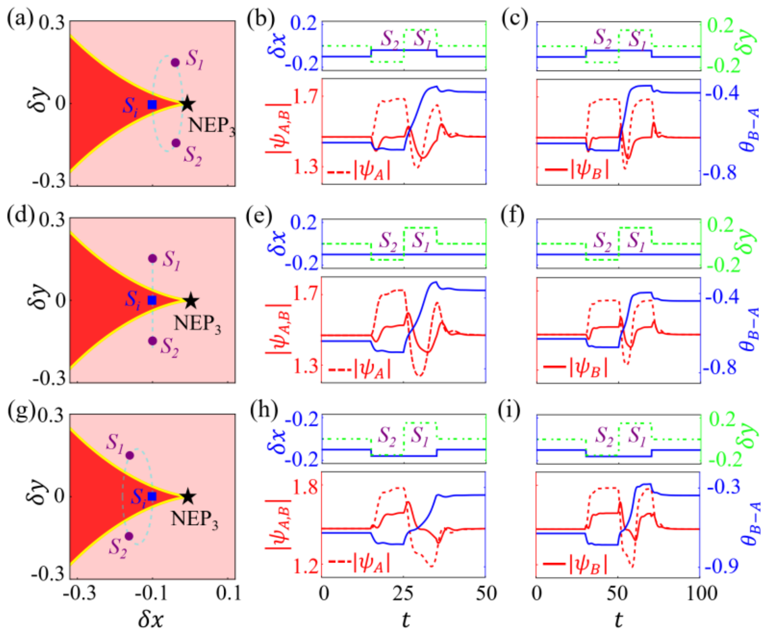

FIG.S7. Reliable predictability in non-adiabatic regimes. Dynamical evolution of the state under different parameter trajectories and evolution periods T. In (a, d, g), we fix $\delta y = 0.15$ and -0.15 for S_1 and S_2, respectively. The values of δx for $\{S_1, S_2\}$ are set to be -0.04 in (a), -0.1 in (d) and -0.16 in (g). Panels (d, e, h) correspond to T=50 and (c, f, i) to T=100. Other parameters used are the same as those in Fig. 2.

Below, based on our experimental design, we show that our NCST scheme still maintains reliable predictability even in non-adiabatic regimes—that is, the NCST remains stable over a reasonably broad parameter range. Following the approach of typical experimental practice[3,19], we first specify representative parameter-evolution trajectories [see the first column of Fig. S7] that correspond to the maximum deviations induced by technical limitations during experiments. We then vary the evolution time to analyze the final state and assess the feasibility of realizing non-adiabatic CST. The second and third columns of Fig. S7 show the evolution trajectory of steering parameters in the parameter space and the states in the phase space for different period (T). The different periods used here represent the maximum evolution-time deviations that may arise due to experimental imperfections. Clearly, NCST can be successfully realized within these non-adiabatic regimes, thereby demonstrating the reliable

predictability required for experimental implementation.

Supplementary Note 6:NCSTs initiated from a higher phase difference stable state.

In this section, we simulate NCSTs starting from a higher phase difference stable state ($\theta_{B-A} = -0.36$). Same as Fig. 2 in the main text, the system starts with an initial state in the bistable region (marked by the blue squares). For a loop centered at the NEP_3 [see Fig. S8(a)], Fig. S8(b, c) shows the evolution trajectory of steering parameters $\{\delta x,\ \delta y\}$ in the parameter space (upper panel), and the field amplitudes $\{|\psi_A|,\ |\psi_B|\}$ and relative phase θ_{B-A} (lower panel) as functions of t. The state evolves towards the initial state in the anticlockwise direction [see Fig. S8(b)] and towards the lower phase difference state ($\theta_{B-A} = -0.64$) in the opposite direction [see Fig. S8(c)], thus exhibiting a NCST between the two stable states. Excluding the NEP_3, the chirality of the process can still be retained without nonadiabatic transitions [see Fig. S8(d-f)]. Similar as Fig. 2(g-i), Fig. S8(g-i) show that NCSTs can be achieved even using only three points $\{S_i, S_1, S_2\}$ on a straight line in the parameter space considering the order of S_1 and S_2.

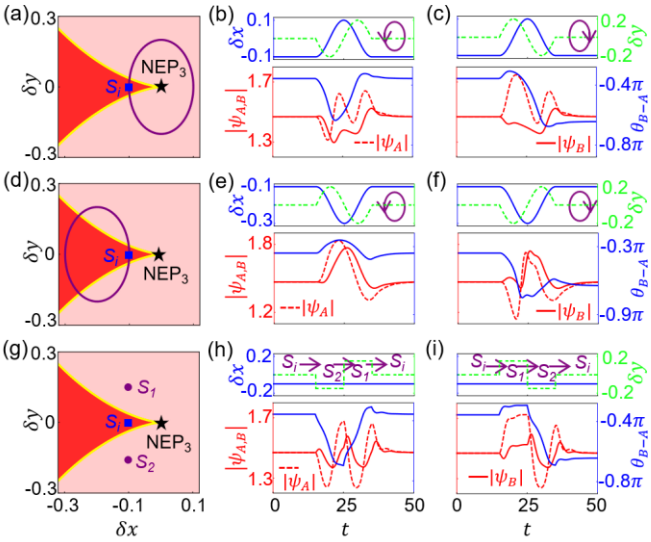

FIG. S8. The same as Fig. 2 in the main text, but starting from a higher phase difference stable state.

Supplementary Note 7: Derivation of the coupled-mode equations.

In this section, we derive Eq. (2) in the main text from Kirchhoff's equations for

the circuit in Fig. S9(a). A similar derivation was also presented in Ref[11]. The system consists of two LC resonators coupled with two capacitors C_c. Voltages V_A and V_B represent the fields inside the left and right resonators, respectively. The nonlinear saturable gain [$-R_A(|V_A|)$, the left dashed block] consists of a voltage amplifier A, a two-diode component D and a series of resistors $\{R_1, R_2, Rg\}$. Here R_2 represents the intrinsic loss rate. Assume that the circuit is working with a time-harmonic field $e^{-i\omega t}$, and Kirchhoff's equations of the circuit are

$$\begin{aligned}\frac{V_A}{-i\omega L_A}-\frac{V_A}{R_A}-i\omega C_0 V_A-i\omega C_c(V_A-V_B)=0,\\ \frac{V_B}{-i\omega L_B}+\frac{V_B}{R_B}-i\omega C_0 V_B-i\omega C_c(V_B-V_A)=0.\end{aligned} \tag{S5}$$

Assuming $C_c \ll C_0$ and $|\omega_{A,B}-\omega| \ll \omega$, where $\omega_{A,B}=1/\sqrt{L_{A,B}C_0}$ represent the resonant frequencies of the uncoupled resonators, then Eq. (1) becomes

$$\begin{pmatrix}\omega_A+i/2C_0R_A & \omega_B C_c/2C_0\\ \omega_B C_c/2C_0 & \omega_B-i/2C_0R_B\end{pmatrix}\begin{pmatrix}V_A\\ V_B\end{pmatrix}=\omega\begin{pmatrix}V_A\\ V_B\end{pmatrix}, \tag{S6}$$

Compared with the tight-binding Hamiltonian in Eq. (2) of the main text, the coupling, loss, and saturated gain terms are given by $\kappa=\omega_B C_c/2C_0$, $l_B=1/2C_0R_B$, and $g_A=1/2C_0R_A$, respectively.

Focusing on the effective gain component [see Fig. S9(b)], the input voltage V_1 at node ① of the operational amplifier is equal to the voltage at node ②. Meanwhile, the current passing through the resistor R_g, $I_{Rg}=\mathrm{V}_1/R_g$, must flow out of node ③ and pass through the diodes. Thus, the voltage on node ③, V_3 is given by

$$V_3=V_1+\frac{\mathrm{V}_1}{R_g}R_D, \tag{S7}$$

Then, the current through the resistor R_1 is given by

$$I_{R_1}=\frac{V_1-V_3}{R_1}=-\frac{\mathrm{V}_1}{R_gR_1}R_D, \tag{S8}$$

Here the "−" sign indicates the current I_{R_1} flows in the direction opposite to the voltage at node ①. Hence from the perspective of the red node in Fig. S9(b), the effective negative resistance (R_n) of the whole system inside the dashed box is $R_n=\mathrm{V}_1/I_{R_1}=-R_gR_1/R_D$. Accordingly, we derive the expression

$$-R_A = -(R_2 R_1 Rg/R_D)/(R_2 - R_1 Rg/R_D). \tag{S9}$$

Figure S9(c) shows the volt-ampere curve of a two-diode (Onsemi BAV99L) composite and the corresponding resistance. The resistance of the two-diode R_D is a monotonic decreasing function of the voltage applied on it. Thus, we can obtain that the gain coefficient $\mathrm{g_A} = 1/2C_0R_A$ decreases with the increase of $|\mathrm{V_A}|$.

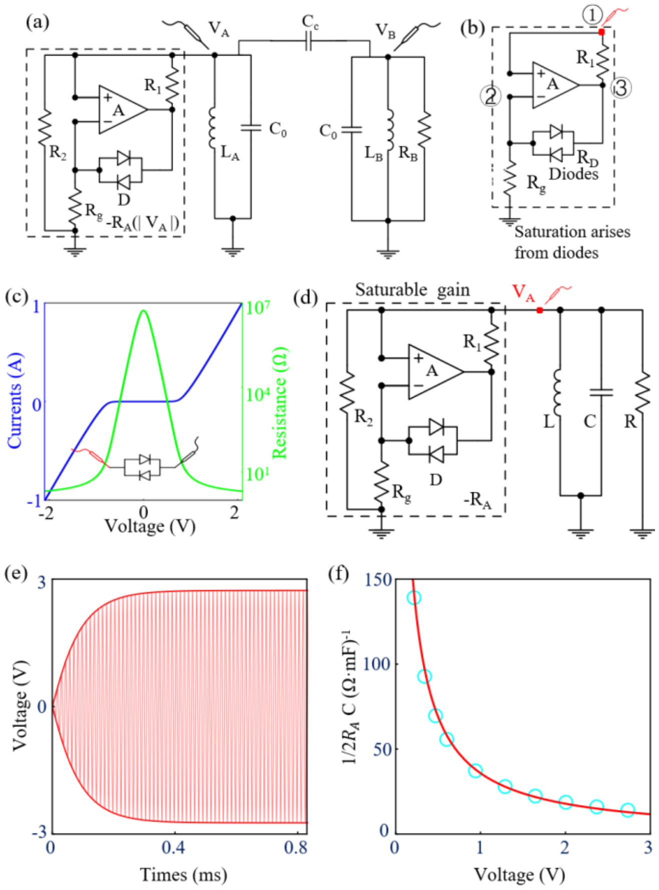

Fig. S9. (a) The circuit used in the experiment, showing the inductors (L), capacitors (C), resistors (R), diodes (D), and an amplifier (A). The left black dashed rectangular marks the negative resistor $-\mathrm{R}_A$ ($|\mathrm{V}_A|$). (b)**,** The nonlinear saturation arises from the two-diodes. (c) The I-V characteristic curve (blue) and the corresponding resistance (green) of the two-diodes composite (Onsemi BAV99L). The inset shows the measurement setup. (d) An LC circuit connected in parallel with a normal resistor R and an effective negative resistor (marked by the black dashed box). (e) Evolution of V_A on the red node in (d) starting with a small kicking-start voltage V_A =1 μV. The solid red lines highlight the envelope. (f) The effective gain $(2R_AC)^{-1}$ as a function

of V_A. The solid line is the fitting curve using Eq. (S11) with fitting parameters: a = 699, b = 18, and c = 1. In (d, e, f), $R_g = 5.1\mathrm{k}\Omega$, $R_2 = 100\mathrm{k}\Omega$, $R_1 = 500\Omega$, $L = 250\mathrm{uH}$, and $C = 18\mathrm{nF}$.

We can also obtain the form of the saturation gain from numerical simulations with the circuit shown in Fig. S9(d). We start the simulations with a small initial voltage (1 μV) on the capacitor to kick start oscillations, and Fig. S9(e) shows the evolution of V_A till a steady state is reached. The corresponding Kirchhoff's equation of the circuit in Fig. S9(d) is

$$\frac{V_A}{-R_A} + \frac{V_A}{-i\omega L} - i\omega C V_A + \frac{V_A}{R} = 0. \tag{S10}$$

The effective resistance $-R_A$ gives the saturable gain of our system. When the voltage reaches a stable value, $R_A = R$ and thus we obtain R_A as a function of the field amplitude (here the voltage at the node A) as shown by the open cyan circles in Fig. S9(f). These data points can be approximated by a fitting curve [the red line in Fig. S9(f)]

$$g(|V_A|) = \frac{1}{2R_A C} = \frac{a}{1 + b|V_A|} - c. \tag{S11}$$

Here, *c* represents the intrinsic loss rate, *a* and *b* are real fitting parameters.

Supplementary Note 8: Configuration of two single-pole-double-throw switches.

In this section, we detail the method for driving the system's evolution among three distinct points in the parameter space using two single-pole-double-throw switches, ADG1519. Figure S10(a) presents the functional block diagram of ADG1519. When the EN pin is set to the high operating voltage VEN (5V in our circuits), the IN logic input determines which switch is turned on. Thus, the voltage V_{IN} at the IN pin, supplied by an arbitrary waveform generator (AWG), controls the conduction paths. Specifically, when V_{IN} is below 0.8V, terminal D and terminal SA are connected; when V_{IN} exceeds 2V, conduction occurs between terminal D and terminal SB.

Inductors $\{L_0, L_1, L_2, L_i\}$ along with two ADG1519 (denoted as U_1 and U_2 respectively) are connected as illustrated in the schematic in Fig. S10(b). By controlling

the voltages at the IN pins of U_1 and U_2 (labeled as V_{IN1} and V_{IN2}, respectively), we are able to determine the conduction pathways, thereby controlling the total inductance L_A. To be concise, in Fig. 3(a) of the main text, we use Fig. S10(c) to illustrate the configuration of the inductors in Fig. S10(b). Table S2 lists the correspondence between the voltages V_{IN1}, V_{IN2}, and the total inductance L_A.

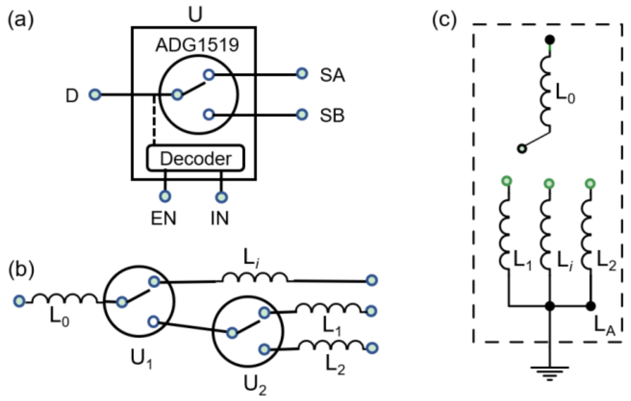

FIG. S10. (a) Functional block diagram of ADG1519. (b) Diagram showing two interconnected ADG1519 switches (labeled as U_1 and U_2), where the voltage at the IN pins of $U_{1,2}$ (V_{IN1} and V_{IN2}) controls the respective conduction pathway. (c) Simplified schematic of the configuration shown in (b). In our setup, the inductances of L_0, L_1, L_2 and L_i are measured as 210.1 uH, 32.2 uH, 0 uH, and 15 uH, respectively, at a working frequency of 70 kHz and a working voltage of 0.5V.

V_{IN1}	V_{IN2}	L_A
Low	Low	$L_0 + L_i$
Low	High	$L_0 + L_i$
High	Low	$L_0 + L_1$
High	High	$L_0 + L_2$

TABLE S2. The correspondence between the voltages V_{IN1}, V_{IN2}, and the total inductance L_A. The first and second columns list the voltages at the IN pins of the switches U_1 and U_2, respectively. The rightmost column gives the total inductance.

Supplementary Note 9: Circuit elements on the PCB.

In this section, we provide details of the circuit elements on the PCB. The upper panel of Fig. S11 shows a photo of the PCB used in our experiments, wherein different functional regions are located roughly according to the circuit in Fig. 3(a) as outlined by the dashed box. The lower panels of Fig. S11 show zoomed-in photos of a few representative elements. From ① to ⑧, they are a single-pole-double-throw (SPDT) switch (ADG1519), an inductor (Murata MDH12577C), a resistor R_g =5.1 kΩ (PTFR0603B5K10N9), a diode (Onsemi BAV99L), an operational amplifier (op-amp) (TI LM7171), a capacitor (Murata GRM21B5C1H183JA01L), a variable resistor (3296W-1-502LF), and a terminal barrier block, respectively. We include a few alternative capacitors, resistors, and inductors for tuning the initial parameters. The terminal barrier blocks have two nodes, each of which can be used for measuring the voltage and connecting the circuit elements as designed.

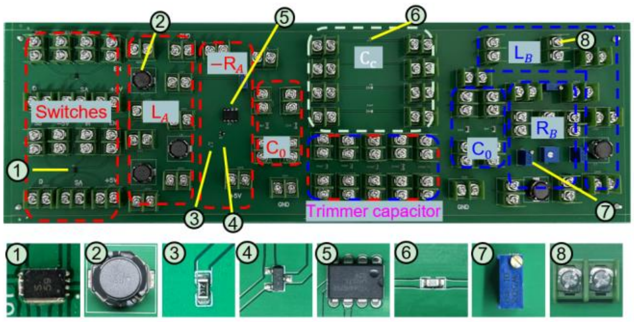

FIG. S11. Details of circuit elements on the PCB. ① single-pole-double-throw (SPDT) switch, ② inductor, ③ resistor, ④ diode, ⑤ operational amplifier (op-amp), ⑥ capacitor, ⑦ variable resistor, ⑧ barrier terminal block.

Supplementary Note 10: Measured relative phases, ratio of voltages and frequencies of stable states

In this section, we provide measured resonance frequencies ω, relative phases θ_{B-A}, and the ratios of voltages $|V_A/V_B|$ for the stable steady states as functions of the inductance L_A. To fine tune L_A, we add a homemade variable resistor [lower left in Fig.

3(b) of the main text, see also Refs. [9,11]] connected in series with a standard inductor (L_0, Murata MDH12577C-221MA) with the inductance measured to be 210.1uH (at 70kHz and 0.5V). To reduce possible errors in the fabrication, the parameters (inductors, capacitors and resistors) in the circuit are measured with a precision LCR meter (TH2829C). Experimental errors in our setup mainly come from two aspects. On the one hand, circuit elements used in experiments, such as capacitors and inductors, are not ideal and are accompanied by possible parasitic parallel resistances, series resistances, parallel shunt capacitances, and series inductances. However, when chosen carefully, the capacitors (Murata GRM21B5C1H183JA01L) and inductors (Murata MDH12577C) can be regarded as capacitors and inductors in series with inherent resistors (labeled as R_C or R_L). On the other hand, the saturable gain consists of a voltage amplifier A, a series of resistors $\{R_1, R_2, R_g\}$ and a two-diodes *D*. This circuit component unavoidably contains unwanted parasitic capacitances, which mainly come from the junction capacitance of the diodes and the parasitic capacitance of the op-amp. Nonetheless, the inherent resistors and unwanted parasitic capacitances can be taken into consideration by an effective shift relative to the measured resistance and capacitance. A more detailed analysis of these experimental errors can be found in the Supplemental Material Sec. 6 of Ref. [[9]].

The markers in Fig. S12 show the measured resonance frequencies ω, voltage ratios $|V_A/V_B|$ and relative phases $(\theta_{B-A} \equiv \theta_B - \theta_A)$ versus L_A in (a-c) and versus δy in (d-f), respectively. Here, the perturbation δy is defined as $\delta y \equiv (\omega_A - \omega_{A0})/2\pi$, where ω_{A0} represents the resonance frequency of resonator *A* when the inductance of L_A is equal to $229.1\ \mu\mathrm{H}$. The lines in Fig. S12 are obtained from numerical simulations by solving Kirchhoff's equations with the solid and dashed lines representing the stable and auxiliary steady states, respectively. The measured values are averaged over 8 independent measurements. The experimental errors (standard deviation) are smaller than the marker size. For demonstration purposes, we exaggerate the error bars by a factor of five.

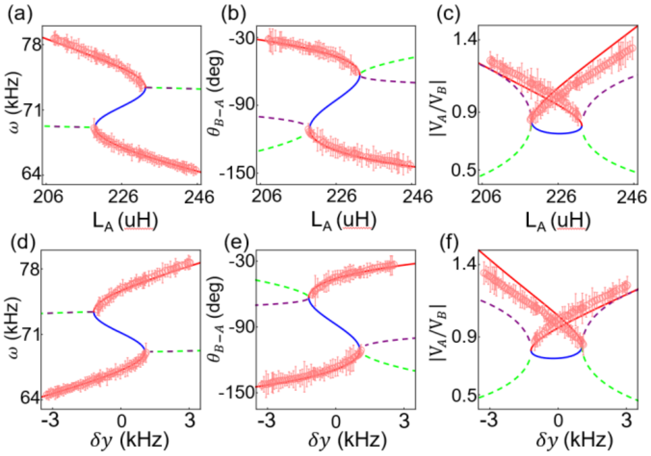

FIG. S12. Measured resonance frequencies (a, d), relative phase (b, e) and voltage ratio (c, f) versus L_A and δy. The values of L_A used in the simulations have been shifted upwards relative to the measured values by 4 μH to compromise for the influence of the parasitic capacitance. And the inherent series resistance is $R_C + R_L = 2.8\ \Omega$. All the other parameters used in the simulations are extracted from the experimental measurements: C_0=18.3 nF, C_c=4 nF, L_B=226.9 uH and R_B=945.1 Ω.

Supplementary Note 11: Evolution trajectories from different initial states.

In this section, we detail the design of the system's initial state and map the evolution of the system to the phase space (θ_{B-A}, $|V_A|$, $|V_B|$). We set the AWG to generate a required external driving signal V_s with amplitude $|V_s|$ and frequency ω_s, and then enforce this signal on the left resonator through a coupling resistor R_e (see the left side of Fig. S13). Figure S13 shows the corresponding circuit model. When the switch S is closed, the corresponding Kirchoff's equations of this circuit are

$$I_{LA} - \frac{V_A}{R_A} + C_0\dot{V}_A + C_c(\dot{V}_A - \dot{V}_B) + \frac{V_A - V_s}{R_e} = 0,$$
$$I_{LB} + \frac{V_B}{R_B} + C_0\dot{V}_B + C_c(\dot{V}_B - \dot{V}_A) = 0, \qquad \text{(S12)}$$

where I_{LA} (I_{LB}) is the current flowing through the inductor L_A (L_B), R_e represents the coupling resistor, V_s is the voltage of this AWG. Taking the approximation same Eq. (4) in main text, Eq. (S12) becomes

$$i\frac{d}{dt}\begin{pmatrix}V_A\\V_B\end{pmatrix}=\begin{pmatrix}\omega_A+\frac{i}{2C_0}\left(\frac{1}{R_A}-\frac{1}{R_e}\right) & \omega_B\frac{C_c}{2C_0}\\ \omega_B\frac{C_c}{2C_0} & \omega_B-\frac{i}{2C_0R_B}\end{pmatrix}\begin{pmatrix}V_A\\V_B\end{pmatrix}+\begin{pmatrix}\frac{i|V_s|}{2R_eC_0}e^{-i\omega_s t}\\0\end{pmatrix} \quad (S13)$$

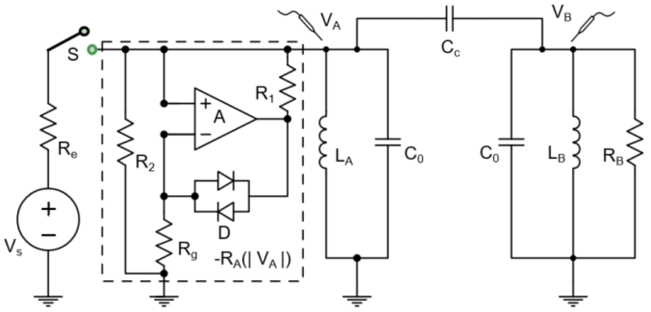

FIG. S13. Circuit used in the experiment to record the dynamics from different initial states. R_e is the coupling resistor, V_s represents the required driving signal which is generated by an AWG.

Since the circuit has an op-amp, after we turn on the circuit, the circuit will automatically reach a stable mode. Then, we apply different external sinusoidal driving signals to resonator A through the coupling resistor R_e and wait until the circuit's operating frequency matches that of the driving signal. After that, we remove the external driving signal and record the relative waveform. Figure S14 (a-c) and Fig. S15 (a-c) show the evolution of V_A (light red lines), V_B (light blue lines) and $V_p \equiv V_A - V_B$ (light green lines) for different driving signals V_s. According to the defintion, $|V_p|$ satisfies the following quation:

$$\begin{aligned}|V_p|e^{-i(\omega t+\phi)} &= |V_A|e^{-i\omega t} - |V_B|e^{-i(\omega t+\theta_{B-A})}\\ &= \sqrt{(|V_A|-|V_B|\cos(\theta_{B-A}))^2+(|V_B|\sin(\theta_{B-A}))^2}\\ &\quad * e^{-i\left(\omega t+\arctan(\frac{|V_B|\sin(\theta_{B-A})}{|V_A|-|V_B|\cos(\theta_{B-A})})\right)}, \end{aligned} \quad (S14)$$

Thus, with the envelope of V_A (red lines), V_B (blue lines) and V_p (green lines) at hand, we can calculate the the phase differences θ_{B-A} from the Eq. (S14). Figure S14 (d-f) and Fig. S15(d-f) show the corresponding evolution of $|V_A|$, $|V_B|$ and θ_{B-A} for different initial driving signals. After removing the driving signal, the V_A and V_B start to oscillate and reaches a stable state within less than 0.5ms. However, the final states reached in Fig. S14 are the lower relative phase states, while those in Fig. S15 are the

higher relative phase states. In other words, in the bistable region, the final state depends strongly on the initial conditions.

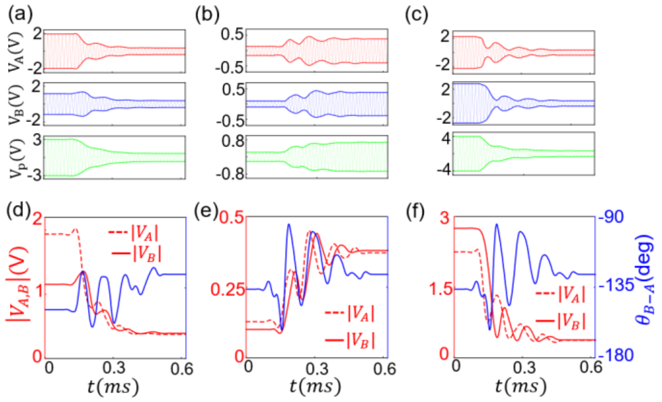

FIG. S14. (a-c) By applying and removing different external driving signal, the red, blue, and green lines show the temporal dynamics of V_A, V_B and $V_p \equiv V_A - V_B$, respectively. The bold red, blue and green lines highlight the corresponding envelope, which remain nearly constant after reaching the stable state. (d-f) From Eq. S14, we can calculate the phase difference θ_{B-A}, which enables us to map the evolution of the system in the phase space (θ_{B-A}, $|V_A|$, $|V_B|$). The driving signal is applied on the circuit till $t \approx 0.09$ ms. Here $R_e = 0.1\ \Omega$, and we apply an external driving signal of 2V and 63 kHz in (a, d), of 0.1V and 65 kHz in (b, e) and of 2.5V and 69 kHz in (c, f), respectively. The other parameters are the same as the Fig. 3(d).

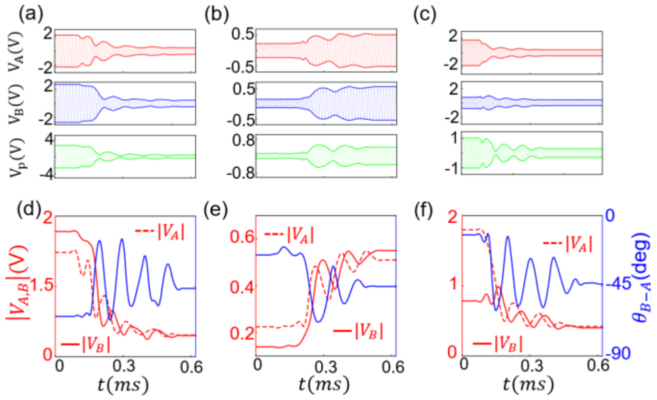

FIG. S15. The same as Fig. S14, but with different external driving signals. Specifically, we apply an external driving signal of 2.5V and 73 kHz in (a, d), 0.2V and 83 kHz in (b, e), and 2V and 90 kHz in (c, f), respectively.

Supplementary Note 12: NCST between bistable states

In this section, we provide supplemental details of the NCST between bistable states observed in our experiments. Figure S16 shows the evolutions of V_{IN1} and V_{IN2} for different trajectories. These voltages are generated by an AWG. When V_{IN} is set as 0 V (<0.8V), conduction occurs between terminal D and terminal SA in Fig. S10(a); when V_{IN} is set as 3V (>2V), conduction occurs between terminal D and terminal SB in Fig. S10(a). Thus, according to Table S1, we can plot the evolutions of δy as shown in the lower panel of Fig. S16.

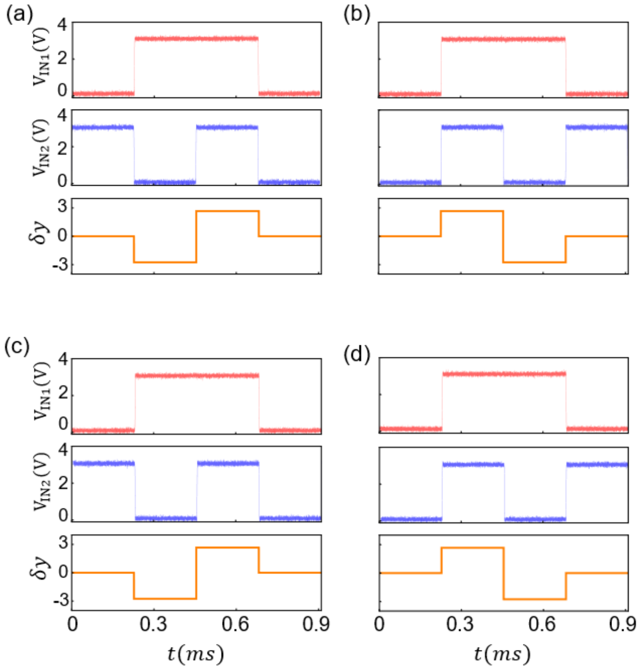

FIG. S16. The evolutions of V_{IN1}, V_{IN2} and the corresponding δy for different initial states and directions of the steering process. Here, V_{IN1} and V_{IN2} are generated by an AWG.

To clearly see the NCST between bistable states, we record the waveforms as shown in Fig. S17. For the direction of the steering process in Fig. S17(a), the state moves towards the higher relative phase state [see Fig. 4(c, e) in the main text]. However, for the opposite direction, as shown in Fig. S17(b) and Fig. 4(b), the state moves towards the lower relative phase state [see Fig. 4(d, f)]. These evolutions

elucidate the fact that regardless of the initial state, the system always moves faithfully towards an eigenstate mandated by the direction of the steering process.

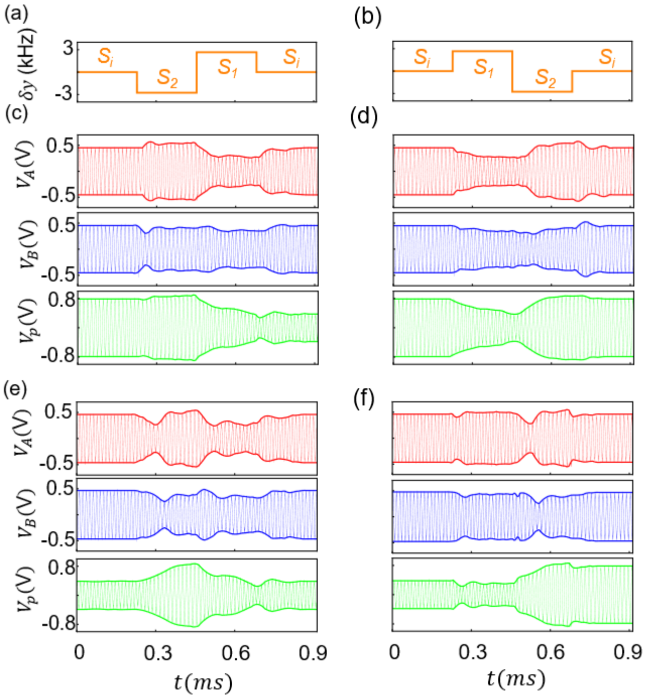

FIG. S17. (a, b) The evolutions of δy for different directions of the steering process. (c-f) The waveforms recorded for the demonstration of the NCST. (c, d) Given the initial state as the lower relative phase state, the state evolves to the higher relative phase state along the direction of (a) and to itself along the direction of (b). (e, f) The similar as (c, d), but taking initial state as the higher relative phase state. Thus, NCST is achieved within only three isolated points in the parameter space. The parameters used are the same as those in Fig. 4.

References

1. Uzdin, R., Mailybaev, A. & Moiseyev, N. On the observability and asymmetry of adiabatic state flips generated by exceptional points. *J. Phys. A Math. Theor.* **44**, 435302 (2011).
2. Hassan, A. U. *et al.* Chiral state conversion without encircling an exceptional point. *Phys. Rev. A* **96**, 1–5 (2017).
3. Nasari, H. *et al.* Observation of chiral state transfer without encircling an exceptional point. *Nature* **605**, 256–261 (2022).
4. Li, A. *et al.* Hamiltonian Hopping for Efficient Chiral Mode Switching in Encircling Exceptional Points. *Phys. Rev. Lett.* **125**, 187403 (2020).
5. Shu, X. *et al.* Fast encirclement of an exceptional point for highly efficient and compact chiral mode converters. *Nat. Commun.* **13**, 2123 (2022).
6. Shu, X. *et al.* Chiral transmission by an open evolution trajectory in a non-Hermitian system. *Light Sci. Appl.* **13**, 65 (2024).
7. Wang, H., Assawaworrarit, S. & Fan, S. Dynamics for encircling an exceptional point in a nonlinear non-Hermitian system. *Opt. Lett.* **44**, 638 (2019).
8. Assawaworrarit, S., Yu, X. & Fan, S. Robust wireless power transfer using a nonlinear parity–time-symmetric circuit. *Nature* **546**, 387–390 (2017).
9. Bai, K. *et al.* Observation of Nonlinear Exceptional Points with a Complete Basis in Dynamics. *Phys. Rev. Lett.* **132**, 073802 (2024).
10. Bai, K. *et al.* Nonlinear Exceptional Points with a Complete Basis in Dynamics. *Phys. Rev. Lett.* **130**, 266901 (2023).
11. Bai, K. *et al.* Nonlinearity-enabled higher-order exceptional singularities with ultra-enhanced signal-to-noise ratio. *Natl. Sci. Rev.* **10**, nwac259 (2023).
12. Zhu, B. F. *et al.* Anomalous single-mode lasing induced by nonlinearity and the non-Hermitian skin effect. *Phys. Rev. Lett.* **129**, 13903 (2022).
13. Smirnova, D., Leykam, D., Chong, Y. & Kivshar, Y. Nonlinear topological photonics. *Appl. Phys. Rev.* **7**, 021306 (2020).

14. Konotop, V. V., Yang, J. & Zezyulin, D. A. Nonlinear waves in PT -symmetric systems. *Rev. Mod. Phys.* **88**, 035002 (2016).

15. Maczewsky, L. J. *et al.* Nonlinearity-induced photonic topological insulator. *Science (80-.).* **370**, 701–704 (2020).

16. Kirsch, M. S. *et al.* Nonlinear second-order photonic topological insulators. *Nat. Phys.* **17**, 995–1000 (2021).

17. Peng, B. *et al.* Parity–time-symmetric whispering-gallery microcavities. *Nat. Phys.* **10**, 394–398 (2014).

18. Chang, L. *et al.* Parity–time symmetry and variable optical isolation in active–passive-coupled microresonators. *Nat. Photonics* **8**, 524–529 (2014).

19. Doppler, J. *et al.* Dynamically encircling an exceptional point for asymmetric mode switching. *Nature* **537**, 76–79 (2016).
